# Cytogenetic and Microdeletions of AZF Regions of Y Chromo-some Studies in Infertile Males in Northwest of Iran

**DOI:** 10.18502/ijph.v49i7.3595

**Published:** 2020-07

**Authors:** Maryam SABOUR TAKANLOU, Seyed Ali RAHMANI, Yadollah AHMADI ASR BADR, Mehrdad PASHAZADEH, Leila SABOUR TAKANLOU

**Affiliations:** 1. Department of Medical Biology, Medical Faculty, Ege University, Izmir, Turkey; 2. Department of Molecular Biology, Faculty of Basic Sciences, Ahar Branch, Islamic Azad University, Ahar, Iran; 3. Department of Urology, Tabriz University of Medical Sciences, Tabriz, Iran; 4. Department of Immunology, Science and Research Branch, Uludag University, Bursa, Turkey

## Dear Editor-in-Chief

Infertility is the main health problem that affects 10%–15% of couples who are incapable to have children. Infertility in men is known to be the reason for about 50% of these cases (1). Male infertility is indeed a several factorials situation in which types of other factors Consist of hormonal disbalance, erectile dysfunction, infections, anti-sperm antibodies, exposure to chemicals and radiation, testicular cancer, and varicocele may be involved (2). Genetic factors can be blamed for only 10% of cases of infertility in men (3). Two of the most important genetic factors are azoospermia factor (AZF) deletions of the Y chromosome that are present in nearly 10%–15% of men with severe spermatogenic imperfections, and Klinefelter’s syndrome, which is the fundamental etiology for 3%–4% of infertile men (4). Meantime, one or numerous chromosomal abnormalities, particularly autosomal aberration (Consist of Robertsonian and balanced translocations) and pericentric and paracentric inversions have been reported in circa 8% of men with severe oligozoospermia (5). Microdeletions of the Y chromosome (Yq microdeletions) form the main cause for primary spermatogenic defects, with an outbreak about of 10% in individuals with non-obstructive azoospermia or severe oligozoospermia, and involve leastwise one of three azoospermia factor (AZF) regions (AZFa, AZFb, and AZFc) (6). Because karyotype tests failure to diagnosis the mentioned microdeletions in about 10%–15% of infertile men with azoospermia or severe oligozoospermia, a combination of a karyotype test and screening for Y chromosome microdeletions is necessary to confirm the presence of such microdeletions. In the present study, we aimed to investigate the occurrence of AZF microdeletions with karyotypic Y chromosome abnormalities in infertile men with azoospermic and oligozoospermic from northwest of Iran, between January 2014 and October 2016 referred to Infertility Center of Alzahra Hospital of Tabriz, Northwest Iran.

The study contained 100 infertile males. Chromosome analysis was performed on peripheral blood lymphocytes according to the standard method. Multiplex PCR assay for microdeletions was performed by using 18 markers of the AZF region of the Y chromosome. The cytogenetic study revealed of 100 cases, 95 had a normal karyotype (46, XY) and chromosomal abnormality in 5 subjects as shown in two of these patients had 47, XXY karyotype. Two cases had 45, XX, rob t(13;14) (q10;q10) and one case had 46, XY[51] / 47, XY; + mar[11]. Four patients having chromosomal abnormality were azoospermic and one patient was oligozoospermia. In this study, 11 subjects (11/100=11%) were detected as having Y chromosome microdeletions. The frequent microdeletions were detected within the AZFc region, with 5 patients (45.45%). Microdeletions in the AZFb+c region were found in three patients (27.27%), one case (9.09%) with AZFb, one case (9.09%) with AZFd microdeletion ve one patients (9.09%) with AZFd+c microdeletion.

This study demonstrated that Y chromosome microdeletion is a major genetic cause of primary male azoospermia. Detection of genetic defects in the Y chromosome can help manage clinical diagnosis, choose the appropriate therapy, and decrease the incidence of genetic disorders. Carriers of karyotypic Y chromosome abnormalities and Y chromosome microdeletions can be transmitted from infertile fathers to their off-spring by ICSI, and genetic counseling is strongly emphasized for infertile men before employment of assisted reproduction techniques. Nevertheless, in infertile males evaluation of the microdeletion(s) in the Y chromosome with a lot of STS markers can determine the expanse of the effects of deletions. Also, assessment more STS markers will help to the reduction of the cost and technical trouble of the procedures of treatment, therefore it will provide routine utilize of Y chromosome microdeletion screening in infertility clinics.
